# COVID-19 vaccination policies under uncertain transmission characteristics using stochastic programming

**DOI:** 10.1371/journal.pone.0270524

**Published:** 2022-07-22

**Authors:** Krishna Reddy Gujjula, Jiangyue Gong, Brittany Segundo, Lewis Ntaimo

**Affiliations:** Wm Michael Barnes ’64 Department of Industrial & Systems Engineering, Texas A&M University, College Station, Texas, United States of America; Padjadjaran University: Universitas Padjadjaran, INDONESIA

## Abstract

We develop a new stochastic programming methodology for determining optimal vaccination policies for a multi-community heterogeneous population. An optimal policy provides the minimum number of vaccinations required to drive post-vaccination reproduction number to below one at a desired reliability level. To generate a vaccination policy, the new method considers the uncertainty in COVID-19 related parameters such as efficacy of vaccines, age-related variation in susceptibility and infectivity to SARS-CoV-2, distribution of household composition in a community, and variation in human interactions. We report on a computational study of the new methodology on a set of neighboring U.S. counties to generate vaccination policies based on vaccine availability. The results show that to control outbreaks at least a certain percentage of the population should be vaccinated in each community based on pre-determined reliability levels. The study also reveals the vaccine sharing capability of the proposed approach among counties under limited vaccine availability. This work contributes a decision-making tool to aid public health agencies worldwide in the allocation of limited vaccines under uncertainty towards controlling epidemics through vaccinations.

## Introduction

COVID-19 caused by the Severe Acute Respiratory Syndrome CoronaVirus (SARS-CoV-2) was declared a global pandemic by the World Health Organization in early 2020. The first reported outbreak occurred in Wuhan, China in December, 2019 and has spread to every region of the world [1]. To control the spread of COVID-19, governments have introduced vaccines and implemented a variety of non-pharmaceutical interventions such as social distancing, restrictions on gatherings, mask mandates, closures of businesses, religious institutions, and schools, travel restrictions and border closures, quarantining, and contact tracing [2–4]. In this paper, we consider a stochastic optimization methodology to determine optimal vaccination policies for a multi-community heterogeneous population to control the spread of the disease. The basic reproduction number, *R*_0_, is used to measure infectious disease community transmission and is defined as the average number of secondary infections caused by a primary case within a completely susceptible population [5, 6]. Under the impact of mitigation measures, the change in transmissibility of the disease over time is evaluated using the *effective reproduction number* *R*_*t*_. It is the average number of secondary infections caused by a primary case at a given time *t* [7, 8]. *R*_*t*_ suggests that an outbreak will continue if it is greater than one and end if it has a value less than one.

In addition to the use of non-pharmaceutical mitigation measures, an effective vaccine can slow down the spread of the disease [9]. Mass vaccination campaigns reduce the susceptible population in a community and can be used to end an outbreak and mitigate any future outbreaks [10]. The reduction in a community’s susceptible population decreases the number of interactions between infectious and susceptible persons, which in turn reduces *R*_*t*_ and will eventually drive *R*_*t*_ to be less than one [10]. Due to the limited availability of vaccines in certain parts of the world, developing an optimized vaccine allocation plan is critical. An optimal allocation would minimize the number of vaccinations required to ensure that *R*_*t*_ ≤ 1. We believe this to be imperative under the following situations: 1) when vaccines are not widely available during the initial stages of distribution [11, 12]; 2) when governments want to reduce the implementation of non-vaccination type mitigation strategies which have a negative impact on socioeconomic activities [13, 14]; 3) when health authorities want to quickly reduce the mortality and morbidity due to COVID-19 in relatively susceptible age groups; 4) when there is vaccine hesitancy, i.e., when a portion of the population is reluctant to receive vaccines [15–17]; and 5) when the emergence of SARS-CoV-2 variants may reduce the efficacy of currently available vaccines.

Mathematical models have been developed to attempt to define an optimal vaccine allocation strategy and these can broadly be categorized as deterministic or stochastic. Deterministic models [18–21, e.g.] include dynamic and optimal control models and do not consider parameter uncertainty. These model are generally sensitive to the vaccine and epidemiological characteristics of the virus. COVID-19 disease characteristics are uncertain at best and studies report values that vary significantly [22, 23]. Thus, it is advantageous to use stochastic modeling approach [24] that account for the uncertainty. Estimating these parameter values, however, is challenging due to the complex nature of human interactions, emerging variants of the virus, as well as the cultural and demographic variability among different communities.

In this work, we build on the stochastic programming (SP) [25, 26] optimal vaccine allocation methodology proposed by [24], which extends the deterministic epidemiology model by [27]. That model was developed to find optimal vaccination policies for a community of households and we extend it to a multi-community framework that considers uncertainties in parameters related to the COVID-19 virus and its vaccines. The SP framework we take accounts for the uncertainty in the model parameters and provides solutions that hedge against unforeseen future scenarios. Unlike solutions obtained from deterministic models using point estimates for the parameters, SP solutions are in fact policies, i.e., decisions prescribed for a given state and/or level of reliability. We implement this multi-community model on a set of neighboring counties, i.e., a population center and its surrounding communities with a sparse population. Generally, the epidemic has a higher likelihood of occurring in a densely populated area due to the average person’s higher number of social contacts [28]. Then the epidemic will eventually propagate to the surrounding communities as a result of inter-community social contacts [29]. The multi-community stochastic model informs a vaccine allocation policy that considers a set of communities together rather than as isolated entities.

The main contribution of this work is an SP based methodology for determining the minimum number of vaccines required to control COVID-19 outbreaks (*R*_*t*_ ≤ 1) through a vaccination campaign in a multi-community setting. At the core of this new methodology is a stochastic disease spread model for an age-based heterogeneous population that considers uncertainty in parameters related to vaccine efficacy, disease transmission characteristics, and human interactions. The model also takes into account demographic variations such as household types and age distribution of the communities to decide optimal allocation of vaccines. In addition to determining optimal vaccine policies, the new methodology addresses potential vaccine shortages and the benefits of vaccine resource sharing among neighboring communities. Another contribution of this work is a computational study based on a real setting that illustrates how the results of this can be used to guide health officials in mitigating epidemics.

The rest of this paper is organized as follows: We derive SP models for vaccine allocation for multiple communities in the next section. We then present population datasets and the uncertain parameters used in the model. Next, we report and discuss the results for two cases: unlimited and limited vaccine availability. We end the paper with concluding remarks and directions for future work.

## Materials and methods

We consider a model of disease transmission in a community based on the work of Becker and Starczak [27] and Tanner et al. [24]. The former work derive a deterministic model of disease transmission which the latter extends to the stochastic setting, where disease transmission parameters are uncertain. Both models consider a single community of households. However, this work extends this approach to multiple communities in a stochastic setting with age-based heterogeneous populations. The aim of a vaccination strategy is to prevent epidemics by immunizing a sufficiently large number of members of a community to force *R*_*t*_ to be less than one. The proportion of individuals in each household that must be vaccinated to prevent an epidemic depends on, among other things, the distribution of household sizes and how the vaccines are allocated to households. In this work, we are interested in determining an optimal strategy for vaccinating members of the community based on household size, given a finite amount of vaccine doses. A vaccination policy or strategy provides *critical vaccination coverage* that reduces *R*_*t*_ to one or less. The term “vaccination coverage” refers to the proportion of individuals who are vaccinated. Our objective is to identify the minimal vaccination coverage.

The critical vaccination coverage for a given vaccination strategy is typically based on the *effective reproduction number* for infected individuals, which is the average number of secondary cases generated by an infected person. We consider the effective reproduction number for infected households in a community under vaccination, which is denoted *R*_*HVc*_ and called the *post-vaccination reproduction number*, as defined by [27]. The aim of a vaccination strategy is to keep *R*_*HVc*_ ≤ 1 to ensure that introductions of the disease do not lead to an epidemic. Therefore, we want to compute the vaccine coverage required so that the vaccine induced herd immunity is at a sufficiently high level to prevent epidemics. We define the nomenclature we use in our mathematical model in Table 1.

**Table 1:** Notation used in defining the models.

**Sets and Indices**	
C	Set of communities, element c∈C.
N	Set of household types, element n∈N.
K	Set of person groups, element k∈K.
V	Set of vaccination policies, element v∈V.
Ω_*c*_	Set of outcomes (scenarios) for community c∈C, element *ω*_*c*_ ∈ Ω_*c*_.
**Parameters**	
$R_{HVc}\left({\tilde{\omega }}_{c}\right)$	Uncertainty post-vaccination reproduction number for community c∈C.
${\tilde{\omega }}_{c}$	Multivariate random variable whose outcome is *ω*_*c*_ ∈ Ω_*c*_ with probability of occurrence $p_{\omega_{c}}$; describes the uncertain parameters for *R*_*HVc*_.
$a_{nvc}\left({\tilde{\omega }}_{c}\right)$	Uncertain *R*_*HVc*_ parameter that captures the impact of vaccination policy v∈V in a type *n* household in community c∈C.
$m_{c}\left({\tilde{\omega }}_{c}\right)$	Uncertain number of close contacts that an infective makes on average with persons from other household in the course of his/her infectious period in a community c∈C.
*H*_*c*_	Number of households in community c∈C.
*p*(*n*)	Number of persons in a household of type *n*.
*f*(*v*)	Number of persons vaccinated in a household when vaccination policy v∈V is implemented.
*h*_*nc*_	Proportion of type *n* households in community c∈C.
*μ*_*c*_	Average household size in a community, $\mu_{c}=\sum_{n\in N}p\left(n\right)h_{nc}$.
*π*_*kc*_	Proportion of group k∈K persons in community c∈C.
$b({\tilde{\omega }}_{c})$	Uncertain transmission rate within a household.
$\beta_{kc}\left({\tilde{\omega }}_{c}\right)$	Uncertain susceptibility for type k∈K person in community c∈C.
$\lambda_{kc}\left({\tilde{\omega }}_{c}\right)$	Uncertain infectivity for type k∈K person in community c∈C.
$\epsilon ({\tilde{\omega }}_{c})$	Uncertain vaccine efficacy.
*V*	Total number of available vaccines.
*α*_*c*_	User-set model reliability level for community c∈C.
${\bar{\alpha }}_{c}^{e}$	Excess allowed on model reliability level for community c∈C.
${\bar{\alpha }}_{c}^{s}$	Deficit allowed on model reliability level for community c∈C.
*M*_*e*_, *M*_*s*_	Sufficiently large numbers.
**Decision Variables**	
*x*_*nvc*_	Proportion of *n* sized households with vaccination policy v∈V implemented in community c∈C.
$\alpha_{c}^{e}$	Excess amount above reliability level *α*_*c*_ level for community c∈C.
$\alpha_{c}^{s}$	Deficit amount below reliability level *α*_*c*_ level for community c∈C.

Assuming a *homogeneous* population model in which we assume there are no significant age-related differences in susceptibility and infectivity of individuals within the communities, Becker and Starczak’s [27] define the expression for *R*_*HVc*_ for a deterministic model of disease spread as follows: Given *x*_*nvc*_ and the proportion of *n*-sized households in which vaccination policy *v* has been implemented, *R*_*HVc*_ for a community *c* is expressed as

$$\begin{array}{c} R_{HVc}=\sum_{n\in N}\sum_{v\in V}a_{nvc}x_{nvc}. \end{array}$$ (1)

The parameter *a*_*nvc*_ for a homogeneous population of communities is defined as given by

$$a_{nvc}=\frac{m_{c}h_{nc}}{\mu_{c}}\left[\left(1-b\right)\left(p\left(n\right)-f\left(v\right)\epsilon \right)+b{\left(p\left(n\right)-f\left(v\right)\epsilon \right)}^{2}+bf\left(v\right)\epsilon \left(1-\epsilon \right)\right],$$ (2)

where *b* is the transmission proportion within a household. When *b* = 0 it means that there is no transmission within a household, whereas when *b* = 1 it means that there is complete transmission in an infected household. Defining *b* in this manner allows us to capture the continuum of transmission rates within households. Next, we give a derivation of the model and provide an extension to the stochastic setting.

The reproduction number for infected households, *R*_*H*_, is given by

$$\begin{array}{c} R_{H}=m_{c}v_{c}, \end{array}$$ (3)

where *v*_*c*_ is the mean size of the household outbreak in community *c* when a randomly selected previously uninfected individual has a close contact with an infective. This model can be traced back to Bartoszyński [6]. Let ${\bar{h}}_{nsc}$ denote the mean size of an outbreak in household of size *n* with *s* susceptible members when the disease is introduced by one of the susceptible being infected from outside the household. Then the probability that the individual contacted is one the susceptibles of the households is *s*/*n*.

Now let *π*_*nc*_ denote the proportion of individuals who belong to a house of size *n*. Also, let *η*_*nsc*_ be the proportion of households of size *n* of whom exactly *s* are initially susceptible. Then we have that

$$\begin{array}{c} v_{c}=\sum_{n}\pi_{nc}\sum_{s=1}^{n}\frac{s}{n}\eta_{nsc}{\bar{h}}_{nsc}. \end{array}$$ (4)

Furthermore, *π*_*nc*_ = *nh*_*nc*_/*μ*_*Nc*_, where *μ*_*Nc*_ is the mean household size. The reproduction number for infected households is

$$R_{H}=\frac{m_{c}}{\mu_{Nc}}\sum_{n}h_{nc}\sum_{s=1}^{n}s\eta_{nsc}{\bar{h}}_{nsc}.$$ (5)

When there is no immunity *s* = *n* and the basic reproduction number, *R*_*HO*_, is given as

$$\begin{array}{c} R_{HO}=\frac{m_{c}}{\mu_{Nc}}\sum_{n}nh_{nc}v_{nnc}. \end{array}$$

To prevent epidemics without vaccinations *R*_*HO*_ ≤ 1. In this study, we assume that *R*_*HO*_ > 1 and we want to use vaccinations to bring *R*_*HO*_ below one. Each vaccination, however, provides immunity with probability *ϵ*, which is the vaccine efficacy. Letting *x*_*nvc*_ be the proportion of *n* sized households with vaccination policy *v* (*v* members vaccinated) implemented in community *c*, then we have that

ηnsc=∑v=n-sn(vn-s)ϵn-s(1-ϵ)v-(n-s)xnvc. (6)

Now consider Eqs 5 and 6, and define

anvc=mchncμNc∑s=n-vns(vn-s)ϵn-s(1-ϵ)v-(n-s)h¯nsc. (7)

Then

$$\begin{array}{c} R_{H}=\sum_{n}\sum_{v=0}^{n}a_{nvc}x_{nvc}. \end{array}$$ (8)

Assuming that ${\bar{h}}_{nsc}=1-b+bs,\;b\in \left[0,1\right]$, where *b* = 0 corresponds to no disease transmission within households and *b* = 1 corresponds to total infection, the expression for *a*_*nvc*_ can be written as follows:

anvc=mchncμNc∑s=n-vns(vn-s)ϵn-s(1-ϵ)v-(n-s)(1-b+bs)=mchncμNc[(1-b)∑s=n-vns(vn-s)ϵn-s(1-ϵ)v-(n-s)+b∑s=n-vns2(vn-s)ϵn-s(1-ϵ)v-(n-s)]=mchncμNc[(1-b)(n-vϵ)+b(n-vϵ)2+bvϵ(1-ϵ)], (9)

where the last expression comes from applying the binomial theorem.

In this study, we extend the expression for *a*_*nvc*_ in Becker and Starczak’s model [27] to the *heterogeneous* and *stochastic setting*, i.e., where disease spread affects specific age groups differently and the disease spread and vaccine parameters are assumed to be unknown. Therefore, we model *a*_*nvc*_ as a random variable, $a_{nvc}\left({\tilde{\omega }}_{c}\right)$, in which an outcome (scenario) *ω*_*c*_ of ${\tilde{\omega }}_{c}$ is given by the triple, *ω*_*c*_ ≔ {*m*_*c*_(*ω*_*c*_), *b*(*ω*_*c*_), *ϵ*(*ω*_*c*_)}, with probability of occurrence $p_{\omega_{c}}$. Consequently, we have that

$$\begin{array}{cc} a_{nvc}\left({\tilde{\omega }}_{c}\right) & =\frac{m_{c}\left({\tilde{\omega }}_{c}\right)h_{nc}}{\mu_{c}}[\left(1-b\left({\tilde{\omega }}_{c}\right)\right)\left(p\left(n\right)-f\left(v\right)\epsilon \left({\tilde{\omega }}_{c}\right)\right)+b\left({\tilde{\omega }}_{c}\right){\left(p\left(n\right)-f\left(v\right)\epsilon \left({\tilde{\omega }}_{c}\right)\right)}^{2}\\ & +b\left({\tilde{\omega }}_{c}\right)f\left(v\right)\epsilon \left({\tilde{\omega }}_{c}\right)\left(1-\epsilon \left({\tilde{\omega }}_{c}\right)\right)]. \end{array}$$ (10)

Therefore, *R*_*HVc*_ is also a random variable and is denoted $R_{HVc}\left({\tilde{\omega }}_{c}\right)$ and is expressed as

$$\begin{array}{c} R_{HVc}\left({\tilde{\omega }}_{c}\right)=\sum_{n\in N}\sum_{v\in V}a_{nvc}\left({\tilde{\omega }}_{c}\right)x_{nvc}. \end{array}$$ (11)

### Heterogeneous population model

In the *heterogeneous population* model, we assume that there are significant age-related differences in the susceptibility and infectivity of individuals in all the communities involved. To capture these differences, we define a set of groups of people K in which susceptibility and infectivity are differentiated by age. We denote the susceptibility and infectivity of group *k* in community *c*, respectively, by $\beta_{kc}\left({\tilde{\omega }}_{c}\right)$ and $\lambda_{kc}\left({\tilde{\omega }}_{c}\right)$. We define three age groups, *A*, *B*, and *C*, as follows: *A* = (*age* ≤ 19), *B* = (20 ≤ *age* ≤ 64), and *C* = (*age* ≥ 65). The number of age groups can be increased based on the real setting. For each household of type *n* with *p*(*n*) members, we must specify the number of persons, *p*_*k*_(*n*) belonging to each of the *three* age groups *A*, *B* and *C*. The possible vaccination policies for a type *n* household are represented by (*f*_*A*_(*v*), *f*_*B*_(*v*), *f*_*C*_(*v*)), the number of household members vaccinated in group *A*, *B*, and *C*, respectively. An example illustration of *p*(*n*) values of 1 and 2 is shown in Table 2.

**Table 2:** Example household types and vaccination policies under heterogeneous population for *p*(*n*) = 1 and *p*(*n*) = 2.

Household Type	Household Size	Household Composition	Total vaccination policies	Possible vaccination policies for a type *n* Household
*n*	*p*(*n*)	(*p*_*A*_(*n*), *p*_*B*_(*n*), *p*_*C*_(*n*))	(*p*_*A*_(*n*) + 1)* (*p*_*B*_(*n*) + 1)* (*p*_*C*_(*n*) + 1)	(*n*, *f*_*A*_(*v*), *f*_*B*_(*v*), *f*_*C*_(*v*))
1	1	(1, 0, 0)	2	(1, 0, 0, 0), (1, 1, 0, 0)
2	1	(0, 1, 0)	2	(2, 0, 0, 0), (2, 0, 1, 0)
3	1	(0, 0, 1)	2	(3, 0, 0, 0), (3, 0, 0, 1)
4	2	(2, 0, 0)	3	(4, 0, 0, 0), (4, 1, 0, 0), (4, 2, 0, 0)
5	2	(0, 2, 0)	3	(5, 0, 0, 0), (5, 0, 1, 0), (5, 0, 2, 0)
6	2	(0, 0, 2)	3	(6, 0, 0, 0), (6, 0, 0, 1), (6, 0, 0, 2)
7	2	(1, 1, 0)	4	(7, 0, 0, 0), (7, 0, 1, 0), (7, 1, 0, 0), (7, 1, 1, 0)
8	2	(0, 1, 1)	4	(8, 0, 0, 0), (8, 0, 0, 1), (8, 0, 1, 0), (8, 0, 1, 1)
9	2	(1, 0, 1)	4	(9, 0, 0, 0), (9, 0, 0, 1), (9, 1, 0, 0), (9, 1, 0, 1)

The heterogeneous model requires know the number of members in a household and to what age group each one belongs. The second column *p*(*n*) in Table 2 gives the household size. For example, for *p*(*n*) = 2 household type *n* = 4, 5, 6, 7, 8, 9. Notice that each household type has different age compositions. For instance, for household type *n* = 4 household composition is (2, 0, 0). This means that there are two individuals in age group A and zero individuals in age groups B and C. For a household type *n* = 3, the household composition is (0, 2, 0), i.e., there are no individuals in age group A, two in age group B, and none in age group C.

Given the proportion of type *n* household with *v* vaccinated members, *x*_*nvc*_, the post-vaccination reproduction number $R_{HVc}\left({\tilde{\omega }}_{c}\right)$ for some community *c* is given by Eq (11). Under the assumption of heterogeneity, the explicit expression for $R_{HVc}\left({\tilde{\omega }}_{c}\right)$ considers the age-stratified groups. Consequently, the uncertain parameter $a_{nvc}\left({\tilde{\omega }}_{c}\right)$ for a heterogeneous population of communities can be defined as follows:

$$\begin{array}{cc} a_{nvc}\left({\tilde{\omega }}_{c}\right)= & \\ & \frac{m_{c}\left({\tilde{\omega }}_{c}\right)h_{nc}}{\mu_{c}}\{\sum_{k\in K}^{}\beta_{kc}\left({\tilde{\omega }}_{c}\right)\lambda_{k}\left({\tilde{\omega }}_{c}\right)[\left(1-b\left({\tilde{\omega }}_{c}\right)\right)\left(p_{k}\left(n\right)-f_{k}\left(v\right)\epsilon \left({\tilde{\omega }}_{c}\right)\right)\;+\\ & b\left({\tilde{\omega }}_{c}\right)f_{k}\left(v\right)\epsilon \left({\tilde{\omega }}_{c}\right)\left(1-\epsilon \left({\tilde{\omega }}_{c}\right)\right)\;+\\ & b\left({\tilde{\omega }}_{c}\right)\sum_{k\in K}\sum_{r\in K}\beta_{kc}\left({\tilde{\omega }}_{c}\right)\lambda_{rc}\left({\tilde{\omega }}_{c}\right)\left(p_{k}\left(n\right)-f_{k}\left(v\right)\epsilon \left({\tilde{\omega }}_{c}\right)\right)\left(p_{r}\left(n\right)-f_{r}\left(v\right)\epsilon \left({\tilde{\omega }}_{c}\right)\right)\}, \end{array}$$ (12)

where $\sum_{k\in K}\beta_{kc}\left({\tilde{\omega }}_{c}\right)\pi_{kc}=1$ and $\sum_{k\in K}\lambda_{kc}\left({\tilde{\omega }}_{c}\right)\pi_{kc}=1$ for each community *c*, which restricts the scale of susceptibility and infectivity.

The impact on vaccine policies caused by different age compositions is captured in Eq 12. The interaction among members of the same age group is calculated using

$$\begin{array}{c} \sum_{k\in K}^{}\beta_{kc}\left({\tilde{\omega }}_{c}\right)\lambda_{k}\left({\tilde{\omega }}_{c}\right)\left[\left(1-b\left({\tilde{\omega }}_{c}\right)\right)\left(p_{k}\left(n\right)-f_{k}\left(v\right)\epsilon \left({\tilde{\omega }}_{c}\right)\right)b\left({\tilde{\omega }}_{c}\right)f_{k}\left(v\right)\epsilon \left({\tilde{\omega }}_{c}\right)\left(1-\epsilon \left({\tilde{\omega }}_{c}\right)\right)\right], \end{array}$$

whereas the interactions of different age groups is captured calculated using

$$\begin{array}{c} \sum_{k\in K}\sum_{r\in K}\beta_{kc}\left({\tilde{\omega }}_{c}\right)\lambda_{rc}\left({\tilde{\omega }}_{c}\right)\left(p_{k}\left(n\right)-f_{k}\left(v\right)\epsilon \left({\tilde{\omega }}_{c}\right)\right)\left(p_{r}\left(n\right)-f_{r}\left(v\right)\epsilon \left({\tilde{\omega }}_{c}\right)\right). \end{array}$$

We should point out that under the homogeneity assumption in population, i.e, $\beta_{kc}\left({\tilde{\omega }}_{c}\right)=1$ and $\lambda_{kc}\left({\tilde{\omega }}_{c}\right)=1$ for all *k*, the parameter $a_{nvc}\left({\tilde{\omega }}_{c}\right)$ reduces to:

$$\begin{array}{cc} a_{nvc}\left({\tilde{\omega }}_{c}\right) & =\frac{m_{c}\left({\tilde{\omega }}_{c}\right)h_{nc}}{\mu_{c}}[\left(1-b\left({\tilde{\omega }}_{c}\right)\right)\left(p\left(n\right)-f\left(v\right)\epsilon \left({\tilde{\omega }}_{c}\right)\right)+b\left({\tilde{\omega }}_{c}\right){\left(p\left(n\right)-f\left(v\right)\epsilon \left({\tilde{\omega }}_{c}\right)\right)}^{2}\;\\ & +b\left({\tilde{\omega }}_{c}\right)f\left(v\right)\epsilon \left({\tilde{\omega }}_{c}\right)\left(1-\epsilon \left({\tilde{\omega }}_{c}\right)\right)], \end{array}$$ (13)

which was derived earlier in Eq (2). The scenario *ω*_*c*_ ∈ Ω_*c*_ of ${\tilde{\omega }}_{c}$ specifies the quintuple, *ω*_*c*_ ≔ {*m*_*c*_(*ω*_*c*_), *b*(*ω*_*c*_), *ϵ*(*ω*_*c*_), *β*_*kc*_(*ω*_*c*_), λ_*kc*_(*ω*_*c*_)}, with probability $p_{\omega_{c}}$.

Computing the post-vaccination reproduction number $R_{HVc}\left({\tilde{\omega }}_{c}\right)$ requires several parameters. Due to the uncertainty in the parameters, not all vaccination coverage choices will necessarily bring *R*_*HVc*_ below one. To accommodate the fact that in reality *R*_*HVc*_ will often exceed one under certain scenarios, we use the chance (probabilistic) constraints SP [30, 31] approach to impose a chance constraint on *R*_*HVc*_ ≤ 1 for each community. A chance constraint requires specifying a *reliability level* *α* ∈ (0.5, 1), which sets the minimum total probability of scenarios that must be satisfied to achieve *R*_*HVc*_ ≤ 1. Mathematically, this is expressed as

$$\begin{array}{c} P\{R_{HVc}\leq 1\}\geq \alpha , \end{array}$$

meaning that *R*_*HVc*_ ≤ 1 holds at least *α* × 100% amount of the time, but not necessarily for every scenario. In other words, the constraint can be violated at most (1 − *α*) × 100% of the time, such as when a certain vaccination policy is ineffective and results in an epidemic.

We are now ready to define the minimum coverage problem using chance constraints SP. We start with a basic formulation in which we assume there is an unlimited number of vaccines available. For some specified reliability level *α*_*c*_ ∈ (0.5, 1), the basic optimal vaccine allocation problem for a heterogeneous population can be formally stated as follows:

$$\text{Min}\;\sum_{c\in C}\sum_{n\in N}\sum_{v\in V}f\left(v\right)h_{nc}x_{nvc}$$ (14a)

$$P\{\sum_{n\in N}\sum_{v\in V}a_{nvc}\left({\tilde{\omega }}_{c}\right)x_{nvc}\leq 1\}\geq \alpha_{c},\;\forall c\in C$$ (14b)

$$\sum_{v\in V}x_{nvc}=1,\;\forall n\in N;\;\forall c\in C$$ (14c)

$$x_{nvc}\geq 0,\;\forall n\in N;\;\forall v\in V;\;\forall c\in C.$$ (14d)

The objective function (14a) determines the minimum vaccination coverage across communities. Constraints (14b) are comprised of the chance constraints requiring that $R_{HVc}\left({\tilde{\omega }}_{c}\right)\leq 1$ to prevent an epidemic for each community at least *α*_*c*_ amount of the time. This reliability level corresponds to the decision-maker’s risk in the sense that this constraint cannot be violated more than 1 − *α*_*c*_ of the time; for *α*_*c*_ proportion of scenarios, epidemics will be prevented. This violation is inevitable if, for example, the vaccine efficacy is not sufficiently large. The outcome (scenario) *ω*_*c*_ that determines the value *a*_*nvc*_(*ω*_*c*_) of the random parameter $a_{nvc}\left({\tilde{\omega }}_{c}\right)$ will depend on the distribution of ${\tilde{\omega }}_{c}$. This problem, then, is generally a nonconvex problem and difficult to solve. However, if ${\tilde{\omega }}_{c}$ is discretely distributed with a finite number of outcomes, the problem can be reformulated as a deterministic equivalent problem using mixed-integer programming (MIP) and then solved using MIP techniques. Furthermore, this basic model is separable, which allows each problem to be solved for each community separately. Constraints (14c) determine the proportion of persons to vaccinate for each household size in each community. Finally, constraints (14d) are non-negativity restrictions.

We extend heterogeneous model (14) to the case with limited vaccine availability *V* and allowing for deviations from the specified *α*_*c*_ level for each community as follows:

$$\text{Min}\;\sum_{c\in C}\sum_{n\in N}\sum_{v\in V}f\left(v\right)h_{nc}x_{nvc}-\sum_{c\in C}M_{e}\alpha_{c}^{e}+\sum_{c\in C}M_{s}\alpha_{c}^{s}$$ (15a)

$$P\{\sum_{n\in N}\sum_{v\in V}a_{nvc}\left({\tilde{\omega }}_{c}\right)x_{nvc}\leq 1\}-\alpha_{c}^{e}+\alpha_{c}^{s}\geq \alpha_{c},\;\forall c\in C$$ (15b)

$$\sum_{v\in V}x_{nvc}=1,\;\forall n\in N;\;\forall c\in C$$ (15c)

$$\sum_{c\in C}\sum_{n\in N}\sum_{v\in V}H_{c}f\left(v\right)h_{nc}x_{nvc}\leq V$$ (15d)

$$\alpha_{c}^{e}\leq 1-\alpha_{c},\;\forall c\in C$$ (15e)

$$\alpha_{c}^{e}\leq {\bar{\alpha }}_{c}^{e},\;\forall c\in C$$ (15f)

$$\alpha_{c}^{s}\leq {\bar{\alpha }}_{c}^{s},\;\forall c\in C$$ (15g)

$$x_{nvc},\alpha_{c}^{e},\alpha_{c}^{s}\geq 0,\;\forall n\in N;\;\forall v\in V;\;\forall c\in C.$$ (15h)

The objective function (15a) determines the minimum vaccination coverage while adjusting for the deviation above and below the specified reliability levels for each community. The chance constraints (15b) now include deviation variables $\alpha_{c}^{e}$ and $\alpha_{c}^{s}$ to adjust the reliability level for each community. The decision-maker’s risk is such that the constraint $R_{HVc}=\sum_{n=1}^{N}\sum_{v\in V}a_{nvc}\left({\tilde{\omega }}_{c}\right)x_{nvc}\leq 1$ can be violated in no more than $1-(\alpha_{c}+\alpha_{c}^{e}-\alpha_{c}^{s})$ proportion of scenarios. Constraints (15c) remain as defined before, while constraint (15d) is added to ensure that the total number of vaccines allocated does not exceed the total number of vaccines available for all communities. This constraint links all the communities and is therefore a complicating constraint, which means that the problem is no longer separable. Constraints (15e) ensure that the deviation above *α*_*c*_ does not exceed the allowable amount 1 − *α*_*c*_ for each community. Constraints (15g) and (15f) limit the deviations based on the user-specified bounds $\alpha_{c}^{e}$ and $\alpha_{c}^{s}$, respectively. Finally, constraints (15h) are non-negativity restrictions on all the decision variables.

### Model parameters

We are now ready to describe the parameters used in the two models. The communities are characterized by the distribution of household types within the community, while the stochastic parameters are represented by discrete probability distributions. For the rest of the model parameters, we created discrete distributions based on the information available for COVID-19 transmission characteristics, historical values for the effective reproduction number, and the advertised efficacy of approved vaccines.

#### Demographic data

**Fig 1:**
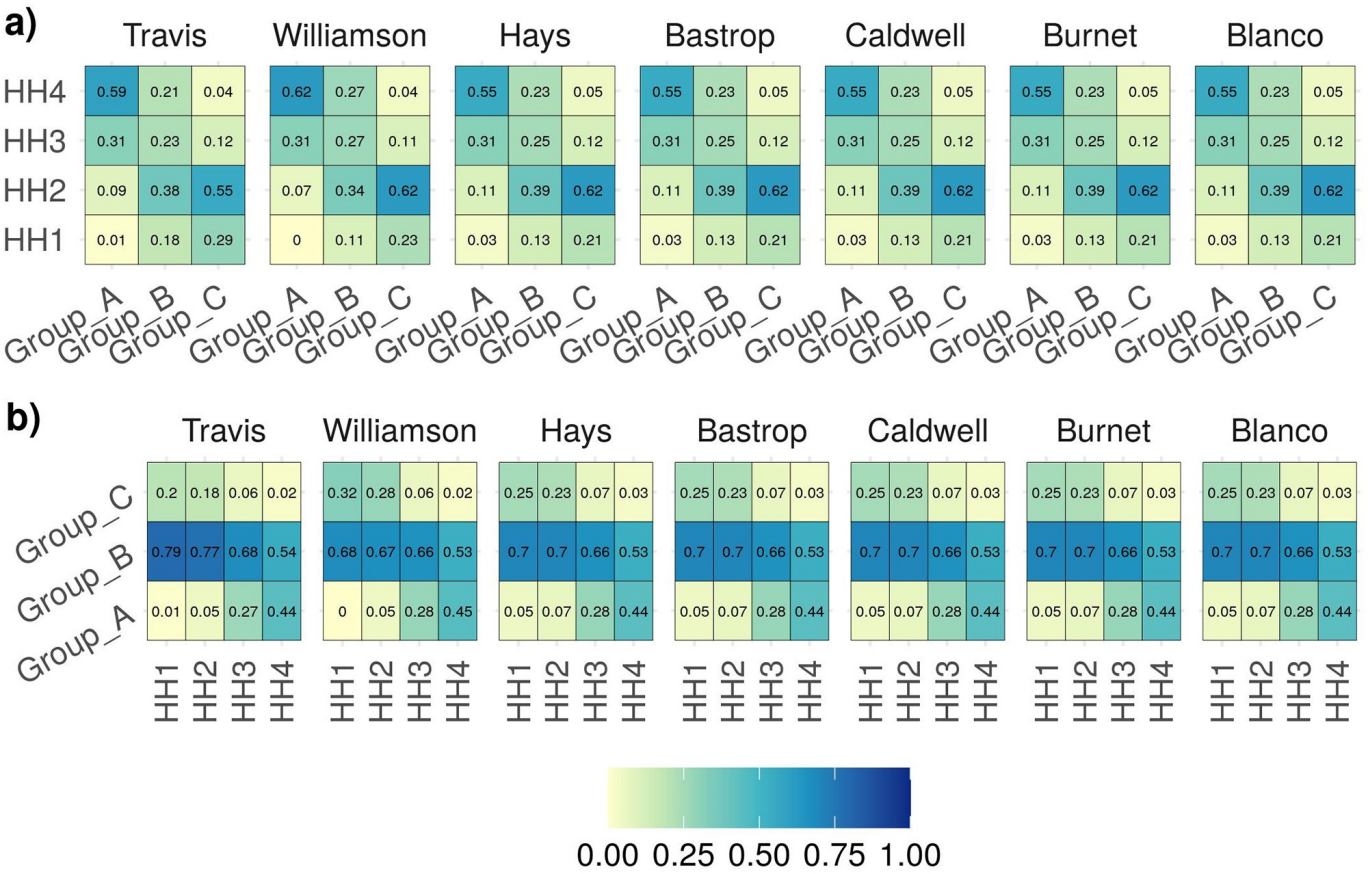
This figure shows the demographic distribution for each county. Fig **a)** shows the heatmap of household sizes in which a particular age group resides. We observe that the majority of the younger population, Group A, tends to belong to larger households along with members of Groups B and C, whereas higher proportion of Group B and C population occupy smaller household of size of one and two. Fig **b)** shows the heatmap of age groups residing in each household size. We observe that smaller households are comprised predominantly of members of Group B population followed by Group C population, and larger households tend to include members of Groups A and B.

We implemented the SP models using data for a multi-community setting comprising seven neighboring counties in the state of Texas, namely; Travis, Williamson, Bastrop, Caldwell, Hays, Burnet, and Blanco. In the model, communities are defined by a multivariate discrete distribution of different household types, which is defined by the size of the household and the number of household members in each age group *A*, *B*, and *C*. We consider household sizes of one to seven and three age groups: *A*: *age* ≤ 19*yrs*., *B*: 20 ≤ *age* ≤ 64*yrs*., and *C*: *age* ≥ 65*yrs*. These age groups are defined on the basis of variation in the effect of COVID-19 on different age groups and can be expanded to include more refined age groups as more information on susceptibility and infectivity for different age groups becomes available. The household size distribution for each of the seven counties was downloaded from 5-year American Survey data (ACS) from (https://data.census.gov/cedsci/) for years 2014–2018 [32]. The household type distribution data was downloaded from https://usa.ipums.org/usa/ [33]. For each county, IPUMS provides a down sampled data along with the weights for each data points. Using the weights, the household type data is scaled up to represent the complete household type distribution for each county. The IPUMS database contains the household data for Travis, Williamson, and Hays counties; for the remaining counties, we assume that the household distribution is similar to that of Hays County. The demographic distribution data utilized in the model is available in S1 File. Fig 1 shows the heat map of the U.S. census demographics data for the different household sizes and age groups.

#### Household transmission rate $b({\tilde{\omega }}_{c})$

This parameter captures the transmissibility of a contagion within a household in a communuty *c*, where $0\leq b({\tilde{\omega }}_{c})\leq 1$. The extreme value $b({\tilde{\omega }}_{c})=0$ is equivalent to no disease transmission within a household, and $b({\tilde{\omega }}_{c})=1$ would mean that all members of the household become infected [27]. This parameter is analogous to household Secondary Attack Rate (SAR), which is defined as the probability that an infected individual will transmit the disease to a susceptible individual [34]. According to various studies, the estimated household SAR varies from 3.9% to 36.4%; when pooled the estimate is 17.1% with a confidence interval (CI) of [13.7%, 21.2%] [35]. Another review estimates that the household SAR value ranges from 4.6% to 49.56% [36]. Based on these values, we generated a discrete distribution (see Table 3) for the within-household transmission rate, $b({\tilde{\omega }}_{c})$.

**Table 3:** The discrete probability distribution represents the within-household transmission rate (*b*) used in this study.

Within-household transmission rate $b({\tilde{\omega }}_{c})$	0.40	0.30	0.20	0.10
Probability	0.10	0.40	0.40	0.10

#### Vaccine efficacy $\epsilon ({\tilde{\omega }}_{c})$

Beginning in December 2020, multiple vaccines were approved for emergency use, and additional vaccines are undergoing vaccine trials and approval. Two of the vaccines approved by the FDA were Pfizer-BioNTech and Moderna, with efficacies of around 95% in clinical trials, and the others vaccines were AstraZeneca-University of Oxford, Johnson & Johnson, and Novavax, which have reported efficacies that range from 60% to 85% [37]. The vaccine efficacy also varies as a result of variability in real-world conditions, such as how the vaccine is transported, how the vaccine is administered, and the medical condition of vaccinated person. Other important factors that may affect the effectiveness of these vaccines include the emergence of new and evasive variants of SARS-CoV-2 and age of the person receiving the vaccine. To complete mass vaccination campaigns, we will need to use multiple vaccine candidates which have different reported and actual efficacies. Under such consideration, a discrete distribution representing vaccine efficacy (see Table 4) is used in the model.

**Table 4:** The discrete probability distribution represents vaccine efficacy ($b({\tilde{\omega }}_{c})$) used in this study.

Vaccine efficacy $\epsilon ({\tilde{\omega }}_{c})$	0.95	0.90	0.80	0.60
Probability	0.20	0.30	0.35	0.15

#### Relative susceptibility $\beta ({\tilde{\omega }}_{c})$

The heterogeneous model assumes age-related differences in susceptibility to COVID-19. *Relative susceptibility* quantifies the variation in susceptibility due to biological susceptibility and social mixing between individuals in different age groups. There is age-dependent variation in susceptibility to COVID-19; studies showed elevated susceptibility in adults over 65 years old and generally lower in the younger population [38, 39]. For each county, we generated three levels of relative susceptibility with associated probabilities (see S1 File). The data for Travis county is shown in Table 5.

**Table 5:** Discrete probability distribution of relative susceptibility $\beta ({\tilde{\omega }}_{c})$ used in this study for Travis county.

Travis county	Age group *A*	Age group *B*	Age group *C*	Scale constraints
Population proportion *π*_*k*_	0.26	0.64	0.10	∑_*k*∈*K*_ *π*_*k*_ = 1.00
Probability	*β*_*A*_	*β*_*B*_	*β*_*C*_	∑_*k*∈*K*_ *β*_*k*_*π*_*k*_
0.50	0.66	1.00	1.91	1.00
0.50	0.80	1.00	1.52	1.00

#### Relative infectivity $\lambda ({\tilde{\omega }}_{c})$

Variation in infectiousness between infected individuals due to differences is biological infectivity and in social mixing between individuals in different age groups is characterized by *relative infectivity*. Goldstein et al. [39] surveyed multiple studies and suggest that there is little evidence that relative infectivity of older age groups is slightly higher than younger population. For infectivity, we assume two cases: *Case 1*. The younger population has lower infectivity compared to the older population (the older population’s biological infectivity outweighs the higher social mixing of the younger population); and *Case 2*. The younger population has higher infectivity compared to the older population (the higher social mixing of the younger population outweighs the older population’s biological infectivity). This nuance is important to the transmission of COVID-19, because the younger population generally has more human interactions [40] and do not develop severe symptoms as compared to older populations. A member of the younger population, then, is more likely to infect a susceptible person. For each county, we generated two levels of relative infectivity with associated probabilities (see S1 File). The data for Travis county is given in Table 6.

**Table 6:** Discrete probability distribution of relative infectivity $\lambda ({\tilde{\omega }}_{c})$ used in this study for Travis county.

Travis county	Age group *A*	Age group *B*	Age group *C*	Scale constraints
Population proportion *π*_*k*_	0.26	0.64	0.10	∑_*k*∈*K*_ *π*_*k*_ = 1.00
Probability (Case 1)	λ_*A*_	λ_*B*_	λ_*C*_	∑_*k*∈*K*_ λ_*k*_*π*_*k*_
0.50	0.95	1.00	1.13	1.00
0.50	0.90	1.00	1.26	1.00
Probability (Case 2)	λ_*A*_	λ_*B*_	λ_*C*_	∑_*k*∈*K*_ λ_*k*_*π*_*k*_
0.50	1.10	1.00	0.74	1.00
0.50	1.15	1.00	0.61	1.00

#### Outside household close contact $m({\tilde{\omega }}_{c})$

An important parameter in the model is $m({\tilde{\omega }}_{c})$, it is defined as the number of close contacts that an infective makes with persons of other households and close contact being sufficient for transmitting the disease when the contact is with a susceptible person. We should point out that $m({\tilde{\omega }}_{c})$ is variable due to differences in human interactions under the impact of various mitigation measures and demographics of a community. To estimate the distribution of $m({\tilde{\omega }}_{c})$, we collected historical time series data for effective reproduction number *R*_*t*_ for Travis county from University of Austin, Texas, COVID-19 dashboard [41]. Using the value of *R*_*t*_ and $a_{nvc}\left({\tilde{\omega }}_{c}\right)$ in Eq 1 we estimate the value of $m({\tilde{\omega }}_{c})$. Note that in Eq 1, *R*_*HVc*_ is analogous to *R*_*t*_, $a_{nvc}\left({\tilde{\omega }}_{c}\right)$ is calculated for homogeneous population, under no vaccination, i.e, vaccine efficacy, $\epsilon ({\tilde{\omega }}_{c})=0$, and point estimate of within household transmission rate, $b({\tilde{\omega }}_{c})=0.3$. Probability associated with the estimated value of *m* is the proportion of time period the value of *R*_*t*_ was observed. *R*_*t*_ values observed in Travis County for the region of Austin metropolitan area varied from 0.5 to 3.5 and for remaining counties from their respective historically observed *effective reproduction number*, *R*_*t*_. The discrete distribution for $m({\tilde{\omega }}_{c})$ is available in S1 File.

#### Reliability level *α*

Typically health officials prescribe acceptable reliability levels based on the historical severity of the epidemic. For this study, we use three levels for reliability: *Low*, *Medium* and *High* (see Table 7). Note that at the highest level of reliability, Travis county has the largest reliability number of 0.990 while the surrounding counties have lower reliability numbers with lowest being 0.955 for Burnet and Blanco counties. These values are set assuming that the epidemic outbreak is more severe at the population centers than in the sparsely populated counties.

**Table 7:** Reliability levels for each community used in this study.

Reliability Level *α*	Travis	Williamson	Hays	Bastrop	Caldwell	Burnet	Blanco
*High*	0.990	0.980	0.970	0.970	0.955	0.955	0.955
*Medium*	0.985	0.975	0.965	0.965	0.950	0.950	0.950
*Low*	0.980	0.970	0.960	0.960	0.945	0.945	0.945

## Results

The stochastic models were implemented in the CPLEX 12.9 Callable Library [42] using C++ and solved using a set of predetermined levels of reliability *α* along with discrete distributions for the model parameters vaccine efficacy, within-household transmission rate, county level household size distribution, and outside-household close contact rate. We solved several instances of the model to generate vaccination policies for the seven Texas counties (Travis, Williamson, Bastrop, Caldwell, Hays, Burnet, and Blanco) based on the all those uncertain parameters. Travis County is the most densely populated of all the seven counties, includes the city of Austin, and is surrounded by the other counties. Due to a lack of extensive studies on age-related differences in infectivity of COVID-19 at the time of this study, we investigated two cases: *Case 1*—Group A has lower relative infectivity than Group C, and *Case 2*—Group C has lower relative infectivity than Group A. We present results for two cases: *unlimited vaccine availability* and *limited vaccine availability*.

### Unlimited vaccine availability

**Fig 2:**
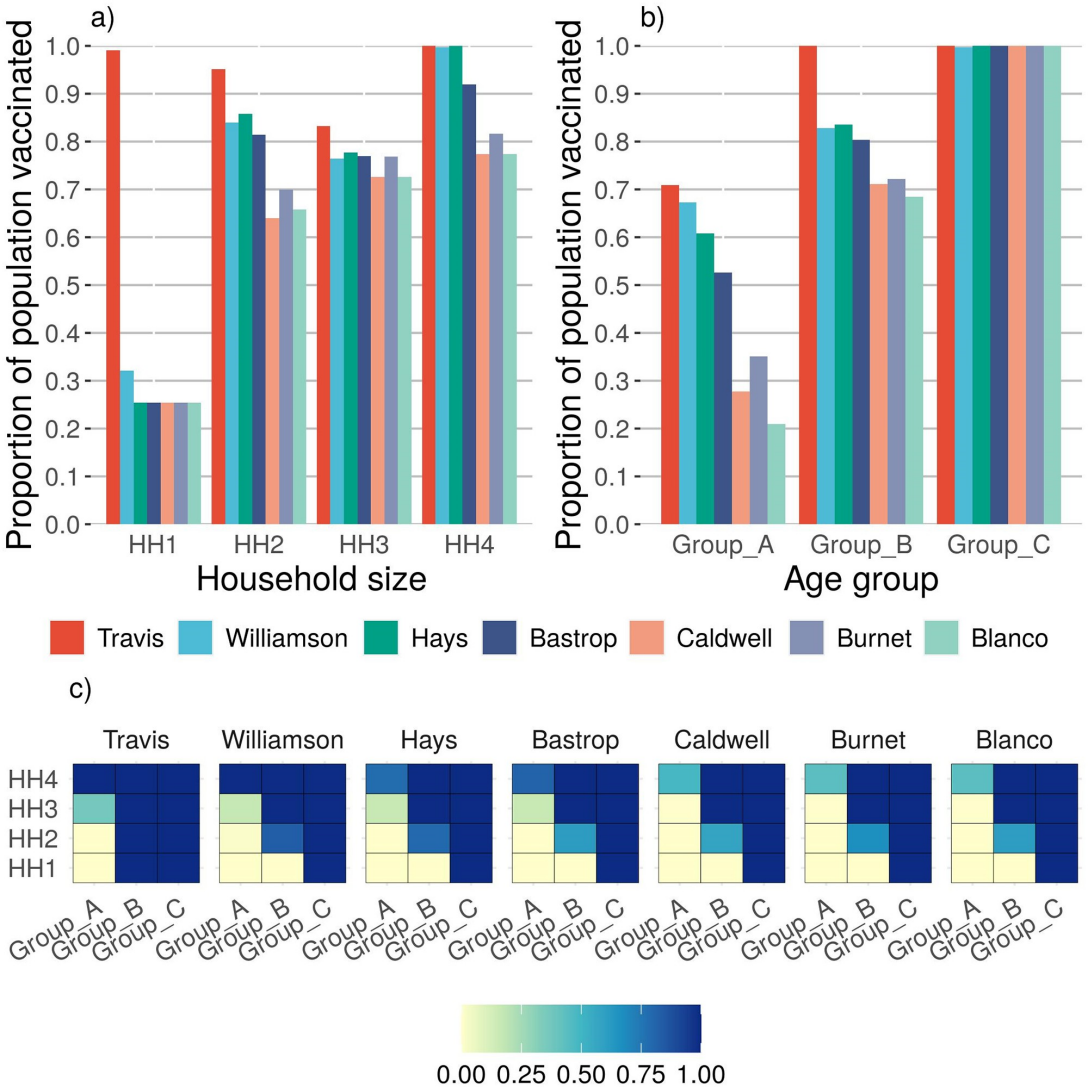
This figure shows the vaccine policy prescribed under the assumption of heterogeneous model for unlimited vaccine availability, under infectivity Case 1 and the *High* reliability level. Fig **a)** depicts the proportion of the population to be vaccinated for each county and household size combination. The figure shows that the higher household sizes tend to be vaccinated at a higher rate. Smaller households of size one and two do have some vaccinations, albeit a lower percentage. These vaccinations are due to the fact that a majority of the population residing in smaller households are from Group B and C. Fig **b)** depicts the proportion of population to be vaccinated by county and age group. The figure illustrates the optimal policy, which is to vaccinate members of Group C population followed by members of Group B and A as a result of the higher relative susceptibility and infectivity for members of Group C. In Fig **c)** a series of heatmaps depict the proportion of population to be vaccinated by the model by household size and age group for each county. The figure indicates that communities should prioritize Group C, followed by Groups B and then A. The priority is to vaccinate Groups B and C, .i.e., populations with higher relative infectivity and susceptibility, and within each population, prioritize members residing in larger households.

**Fig 3:**
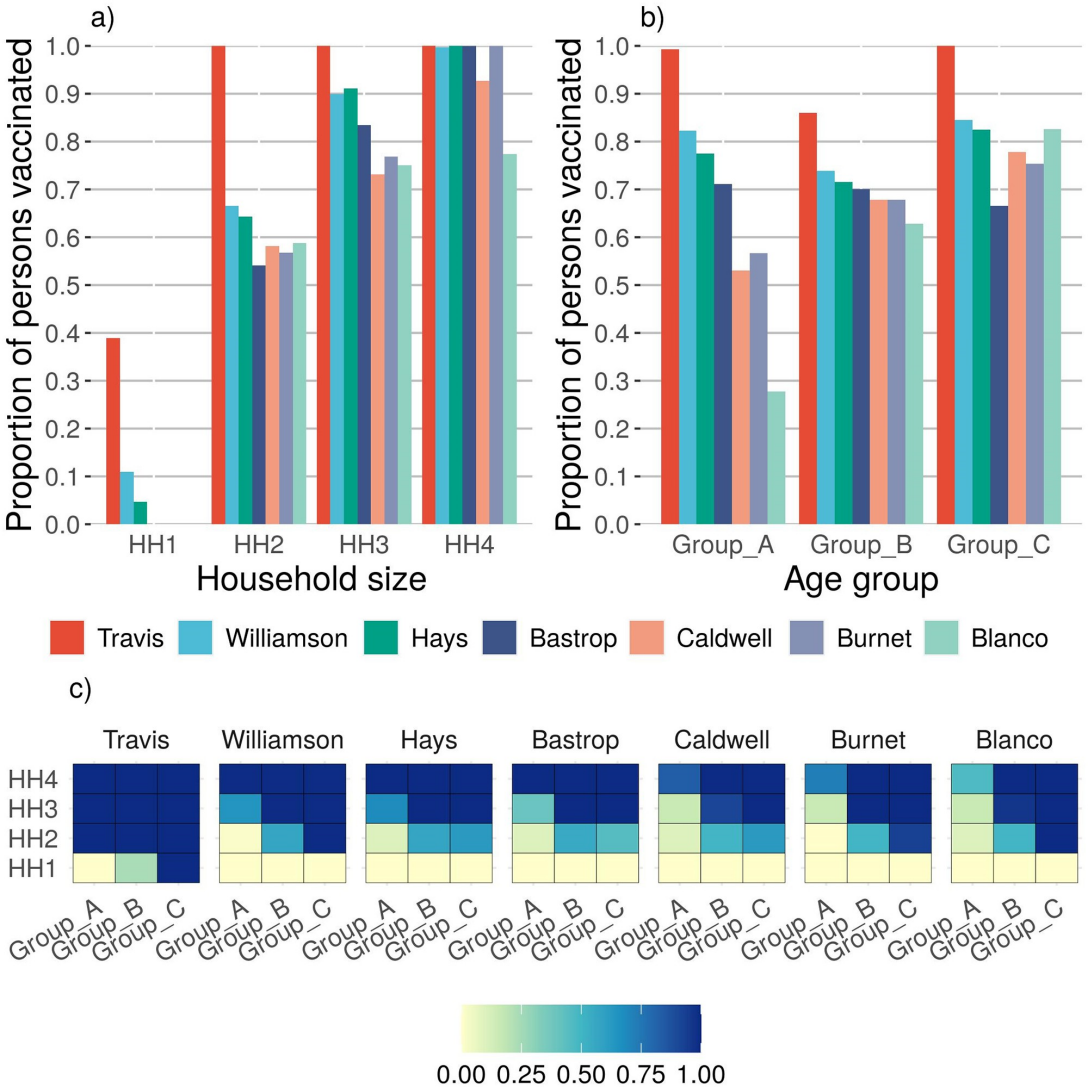
This figure illustrates the vaccination policy prescribed by the heterogeneous model for unlimited vaccine availability, under infectivity Case 2 and *High* reliability. Case 2 is defined such that Group A has a higher infectivity than Group C. **a)** The figure depicts the proportion of the population to be vaccinated by the model by county and household size. The figure shows that communities should vaccinate the larger households at a greater rate. This trend is due to the fact that a large number of members of Group A reside with members of Group B and C, and so a higher vaccination rate is required in this situation in order to effectively block the contagion transmission from a group of higher infectivity to a group of higher susceptibility. **b)** Fig depicts the proportion of population to be vaccinated by county and age group. The figure shows that the optimal policy recommend vaccinating the higher infectivity population, Group A, and the higher susceptibility population, Group C in Travis, Williamson and Hays counties. For Caldwell, Burnet and Blanco the priority is given to Group C followed by Group B and A respectively. The heatmaps in figure **c)** depict the proportion of the population to be vaccinated by household size and age group. The heatmaps illustrate the trend to vaccinate larger households first, and for smaller households, the preference is to vaccinate Group C followed by Group B.

We show the results the three levels of reliability *α*, the two cases of relative infectivity, and the assumption of unlimited vaccine availability. The results are summarized in Table 8. For reliability level *High* and infectivity Case 1, the proportions of the population to be vaccinated to control the epidemic for Travis, Williamson, Hays, Bastrop, Caldwell, Burnet and Blanco counties are 0.94, 0.81, 0.75, 0.71, 0.65, 0.67 and 0.65, respectively. The average proportion of the population across all counties to be vaccinated is 0.87. Observe that when the reliability level is decreased, the proportion of the population to be vaccinated decreases. We observe a similar trend for infectivity Case 2. The vaccination policies prescribed under the assumption of heterogeneous model for unlimited vaccine availability, under infectivity Case 1 and the *High* reliability level are plotted in Fig 2. Those for infectivity Case 2 are plotted in Fig 3.

**Table 8:** The minimum number of vaccinations required to bring *R*_*HVc*_ ≤ 1 under unlimited vaccine availability for heterogeneous populations. The proportion of the population to be vaccinated is in parentheses.

Reliability Level *α*							
Case 1	Travis	Williamson	Hays	Bastrop	Caldwell	Burnet	Blanco
*High*	831153 (0.94)	297649 (0.81)	106120 (0.75)	34837 (0.71)	16694 (0.65)	22394 (0.67)	5383 (0.65)
*Medium*	776772 (0.88)	283034 (0.77)	106451 (0.75)	34771 (0.71)	16573 (0.65)	22297 (0.66)	5322 (0.64)
*Low*	710316 (0.80)	281390 (0.77)	99689 (0.70)	32232 (0.66)	15044 (0.59)	20325 (0.60)	4895 (0.59)
Case 2							
*High*	795161 (0.90)	277961 (0.76)	100010 (0.71)	32603 (0.67)	15713 (0.61)	20748 (0.62)	4925 (0.59)
*Medium*	757667 (0.86)	267998 (0.74)	99488 (0.70)	32470 (0.67)	15474 (0.60)	20426 (0.61)	4836 (0.58)
*Low*	678574 (0.77)	264651 (0.72)	94743 (0.67)	31107 (0.64)	14901 (0.59)	19716 (0.59)	4771 (0.57)

### Limited vaccine availability

**Fig 4:**
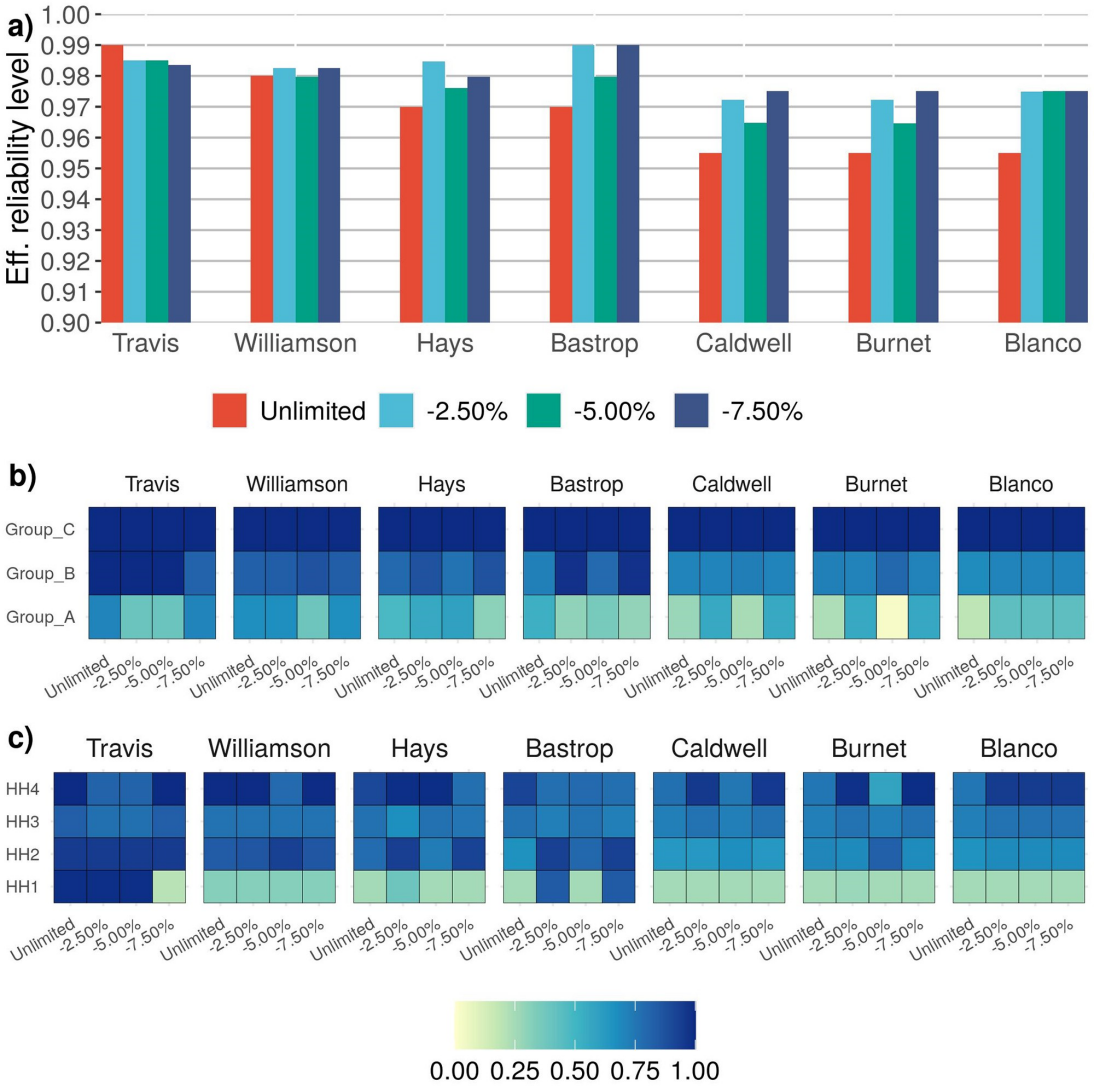
This figure illustrates the reliability adjustment and vaccine sharing capability prescribed by the heterogeneous model under infectivity Case 1, limited vaccine availability and *High* reliability level. **a)** The bar-plot illustrates that reliability adjustment feature of the heterogeneous model under limited vaccination. For *Unlimited* vaccine availability the model achieves the required *High* reliability but as the vaccine availability reduces the reliability levels are adjusted to achieve an optimal vaccination policy. Generally, Travis county reliability is reduced and for other counties reliability is increased. **b)** For each county, the heatmap illustrates proportion of population to be vaccinated within an age Group for a case of vaccine availability. It shows that for Case 1 of infectivity if the reliability is lowered and vaccines are released from a county and assigned to counties where additional reliability is achieved, generally vaccines are first released from Group A followed by Group B. And the counties receiving these additional vaccines assign them to Group C first followed by Group B. **c)** For each county, the heatmap illustrates proportion of population to be vaccinated within a household size for a case of vaccine availability. Across all the counties there is no clear pattern but the plot shows the vaccination policy per household size within a county.

**Fig 5:**
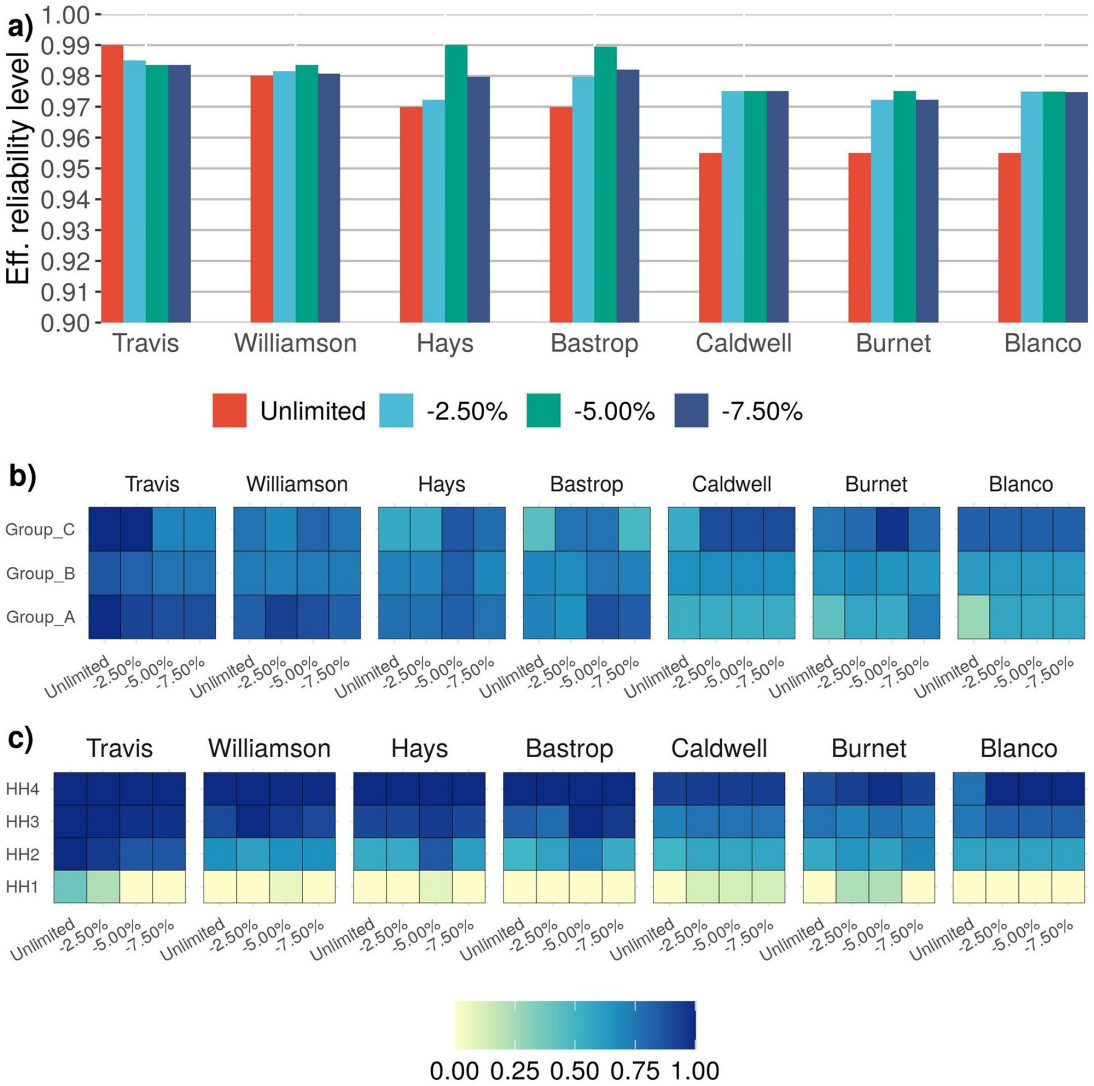
This figure illustrates the reliability adjustment and vaccine sharing capability prescribed by the heterogeneous model under infectivity Case 2, limited vaccine availability and *High* reliability level. **a)** The bar-plot illustrates that reliability adjustment feature of the heterogeneous model under limited vaccination. For *Unlimited* vaccine availability the model achieves the required *High* reliability but as the vaccine availability reduces the reliability levels are adjusted to achieve an optimal vaccination policy. Generally, Travis county reliability is reduced and for other counties reliability is increased. **b)** For each county, the heatmap illustrates proportion of population to be vaccinated within an age group for a case of vaccine availability. Across all the counties there is no clear pattern but the plot shows the vaccination policy per age group within a county. **c)** For each county, the heatmap illustrates proportion of population to be vaccinated within a household size for a case of vaccine availability. It shows that for Case 2 of infectivity if the reliability is lowered and vaccines are released from a county and assigned to counties where additional reliability is achieved, generally vaccines are first released from HH1 followed by HH2. And the counties receiving these additional vaccines assign them to higher household size of three and four.

We also considered the situation in which vaccines are available in limited quantity. The results are provided for the two infectivity scenarios and vaccine shortages of 2.5%, 5.0%, 7.5%. Fig 4 shows and describes the reliability adjustment and vaccine sharing capability prescribed by the heterogeneous model under infectivity Case 1, limited vaccine availability and *High* reliability level. The reliability adjustment and vaccine sharing capability prescribed by the heterogeneous model under infectivity Case 2, limited vaccine availability and *High* reliability level are shown and described in Fig 5. Due to space limitations, complete results for infectivity Case 1 are given in S2 File, and for infectivity Case 2 in S3 File.

## Discussion

Recall that in the heterogeneous model the household types are defined by the size of the household as well as the age groups of the members residing in the household. Therefore, the vaccination policy generated by the heterogeneous model is dependent on both the size and age composition of the members residing in the household. When we consider infectivity Case 1, the results indicate that Group C, which has higher relative susceptibility and infectivity than Group A and B, should be prioritized. The model prefers to vaccinate Group C irrespective of the size of household in which they reside. Group C is followed by Group B and then Group A in terms of the proportion of people vaccinated, where Group A has the lowest relative susceptibility and infectivity (refer to Fig 2b and 2c). With respect to household size, the model recommends to vaccinate similar proportion of inhabitants in households of size two ore more (refer to Fig 2a). This trend occurs because the majority of households of size two and larger tend to have more members of the Group B and C populations than members of Group A (refer to Fig 1b), and, in this case. Groups B and C have higher susceptibility and infectivity than Group A. In short, the model prioritizes vulnerable age groups and, within each group, prioritizes members of larger households (see Fig 2c). This vaccination policy is similar to the one implemented in the U.S. at the early stages of vaccine roll out, in which priority was given to the oldest age group. At the time of this study, the majority of what we call Group A was not eligible for vaccination, because the FDA had not approved the vaccines for members of the population 16-years-old and younger.

Under infectivity Case 2, although the required number of vaccinations is similar to that of Case 1, the model suggests a different vaccination policy. In this case, we assume that the relative infectivity of Group A is higher than that of Group C, and the relative susceptibility of Group A is lower that that of Group C. If we observe the household composition by age, the majority of the people in Group A tend to reside with members of Groups B and C population in larger households of three or more (refer to Fig 1a). In a larger household, the risk of transmission between a member of Group A and a member of Group B or C is higher. As a result, the counties should prioritize members of larger households for vaccination (refer to Fig 3a). Referring to Fig 3b), the model results show that for Travis, Williamson, and Hays counties a higher proportion of Group A and C are vaccinated when compared to Group B. In scarcely populated counties of Caldwell, Burnet and Blanco, the priority for vaccination is given to Group C followed by Group B and Group A, respectively. Fewer members of Group A reside with members of Group B or C in one- and two-person households, so the optimal vaccine policy prescribes fewer vaccinations for smaller households. Unlike Case 1, in Case 2 the solution indicates prioritizing members residing in larger household sizes, and then prioritizing vulnerable age groups—in this case Groups B and C (refer to Fig 3c). The results from this case become more relevant as younger members of the population become eligible for the vaccine.

In the case of vaccine shortages, some counties are unable to achieve the defined reliability levels, and, as a result, the results suggest lower *effective* reliability levels for Travis County, Williamson and Hays counties. However, the model increases the reliability levels for Bastrop, Blanco, Caldwell, and Burnet counties. The increased reliability suggests that additional vaccines can be transferred to these counties in order to mitigate additional outbreak scenarios. For infectivity Case 1, the vaccine policy indicates that instead of vaccinating Group A in the counties where reliability is lowered, those vaccines can be distributed to members of Group C, followed by members of Group B in counties where it is possible to satisfy additional scenarios (see Fig 4). In infectivity Case 2, the solution indicates that vaccines for smaller households in the counties where reliability cannot be met should be redistributed to larger households in counties where additional reliability can be achieved (see Fig 5).

## Conclusion

We introduce a multi-community household-based SP methodology for generating an optimal vaccination policy to control the outbreak of COVID-19. While generating a vaccination policy, this new methodology considers uncertainty inherent in both COVID-19 and human interactions. We develop two stochastic models that use the demographic structure of households based on census data, as well as age-related heterogeneity to COVID-19 in the sub-populations of each community. The models generate vaccination policies under unlimited and limited vaccine availability, respectively, and incorporates the idea of vaccine sharing between communities in order to control COVID-19 outbreaks. The model was implemented and tested based on seven neighboring counties in the U.S. state of Texas. Computational results show that to control the outbreak at least a certain percentage of the population in each county should be vaccinated, depending on the pre-determined reliability levels. The study also reveals the vaccine sharing capability of the proposed model among the counties under limited vaccine availability. This work contributes a new decision-making tool to aid public health agencies in the optimal allocation of vaccines under uncertainty for multiple communities to control epidemics through vaccinations. Future research along this line of work include extending the proposed methodology to include refined age classes and the vaccination status of individuals. Another direction is to include new models to estimate the outside household contact rate and incorporating the logistics of vaccine delivery.

## Supporting information

S1 FileThis file contains the discrete distribution for $m({\tilde{\omega }}_{c})$, which is the number of close contacts that an infective makes with persons of other households.(XLSX)

S2 File This file contains computational results for infectivity Case 1. (XLSX)

S3 File This file contains computational results for infectivity Case 2. (XLSX)
